# Zero-Inflated gaussian mixed models for analyzing longitudinal microbiome data

**DOI:** 10.1371/journal.pone.0242073

**Published:** 2020-11-09

**Authors:** Xinyan Zhang, Boyi Guo, Nengjun Yi

**Affiliations:** 1 Department of Statistics and Data Analytics, Kennesaw State University, Kennesaw, GA, United States of America; 2 Department of Biostatistics, University of Alabama at Birmingham, Birmingham, AL, United States of America; University of Minnesota Twin Cities, UNITED STATES

## Abstract

**Motivation:**

The human microbiome is variable and dynamic in nature. Longitudinal studies could explain the mechanisms in maintaining the microbiome in health or causing dysbiosis in disease. However, it remains challenging to properly analyze the longitudinal microbiome data from either 16S rRNA or metagenome shotgun sequencing studies, output as proportions or counts. Most microbiome data are sparse, requiring statistical models to handle zero-inflation. Moreover, longitudinal design induces correlation among the samples and thus further complicates the analysis and interpretation of the microbiome data.

**Results:**

In this article, we propose zero-inflated Gaussian mixed models (ZIGMMs) to analyze longitudinal microbiome data. ZIGMMs is a robust and flexible method which can be applicable for longitudinal microbiome proportion data or count data generated with either 16S rRNA or shotgun sequencing technologies. It can include various types of fixed effects and random effects and account for various within-subject correlation structures, and can effectively handle zero-inflation. We developed an efficient Expectation-Maximization (EM) algorithm to fit the ZIGMMs by taking advantage of the standard procedure for fitting linear mixed models. We demonstrate the computational efficiency of our EM algorithm by comparing with two other zero-inflated methods. We show that ZIGMMs outperform the previously used linear mixed models (LMMs), negative binomial mixed models (NBMMs) and zero-inflated Beta regression mixed model (ZIBR) in detecting associated effects in longitudinal microbiome data through extensive simulations. We also apply our method to two public longitudinal microbiome datasets and compare with LMMs and NBMMs in detecting dynamic effects of associated taxa.

## 1. Introduction

Since birth, the human body becomes host to millions of microbiota that influence health across whole lives and potentially over generations [1]. The combination of microbiota and the associated genomes (metagenome) interact with the host environment to form the human microbiome [2]. Recent studies have investigated static associations between the human microbiome and many human diseases such as obesity, diabetes, inflammatory bowel disease, irritable bowel syndrome, vaginosis and even cancers [2–7]. However, the microbes could interact with the host and the environment over time [8]. Thus the human microbiome is variable and dynamic in nature, and the infant microbiome could possibly have subsequent implications in future health through the human host’s early life and even adulthood [9]. Longitudinal studies could explain the mechanisms in maintaining the microbiome in health or causing dysbiosis in disease [10]. Recent microbiome studies have employed the longitudinal study design to investigate the dynamic changes of microbial abundance over time and the associations between the microbiome and host environmental/clinical factors [11–15].

As a result of the research interests and the development of high-throughput metagenomics, a large amount of longitudinal 16S rRNA data or metagenome shotgun sequencing data has been generated [16]. It is known that 16S rRNA data or metagenome shotgun sequencing data are both processed and output as number of fragments or reads (in terms of raw or relative abundance) in operational taxonomic units (OTUs) or functional units with various bioinformatics pipelines, such as QIIME and mothur for 16S rRNA data and MetaPhlAn, PhyloSift, and Kraken for shotgun libraries [16]. Although some of the pipelines output the microbiome data in raw counts, others, such as MetaPhlAn, output the relative abundance from shotgun data in proportions.

However, it remains challenging to properly analyze and interpret the longitudinal microbiome data, especially in terms of proportion. Due to both biological and technical reasons, microbiome sequencing data is sparse [17]. Moreover, longitudinal microbiome data possesses special features, for example, time-dependent effects and correlations among the samples within the subjects, for which tailored statistical methods are required [10]. La Rosa, Warner [12], as several previous studies, used linear mixed models (LMMs) to account for correlations in longitudinal microbiome studies [12,18–21]. However, using LMMs is not capable to correct for excess zeros in microbiome data. Recently, we have developed negative binomial mixed models (NBMMs) for analyzing longitudinal microbiome count data, but have not explicitly modeled zero-inflation [22,23]. Romero, Hassan [24] used zero-inflated negative binomial mixed-effects models to analyze longitudinal count data. Neither NBMMs nor the zero-inflated negative binomial mixed-effects models is applicable in analyzing longitudinal microbiome proportion data. Alternatively, Chen and Li [25] proposed a zero-inflated Beta regression model with random effects (ZIBR) for analyzing longitudinal microbiome proportions. However, according to the manual of R package **ZIBR** [26], ZIBR cannot handle missing data, which means each subject must have the same number of time points. Moreover, these two zero-inflated methods have not been developed to account for within-subject correlations and may be computationally sub-optimal for analyzing many OTUs. Thus, statistical models are needed to account for sample correlations over time as well as zero-inflation and other properties of microbiome data [25,27,28].

We here propose zero-inflated Gaussian mixed models (ZIGMMs) and an efficient algorithm to address the previous limitations. Our method is robust and flexible and can analyze longitudinal microbiome proportion data and count data generated with either 16S rRNA or shotgun sequencing technologies. The proposed model can effectively deal with zero-inflation and can include various types of fixed and random effects and within-subject correlation structures. We develop an efficient Expectation-Maximization (EM) algorithm to fit the ZIGMMs by taking advantage of the standard procedure for fitting LMMs. We show computational efficiency of ZIGMMs compared with the other two zero-inflated methods, ZIBR and zero-inflated negative binomial mixed models implemented in the R package **glmmTMB**. Extensive simulations demonstrate that our ZIGMMs outperform the various previously used methods in detecting associated effects in longitudinal microbiome data. We also apply our method to a shotgun longitudinal microbiome proportion data and a 16S rRNA microbiome count data in detecting dynamic effects of associated taxa. We have implemented the ZIGMMs in the R package **NBZIMM**, which is freely available from the public GitHub repository http://github.com//nyiuab//NBZIMM.

## 2. Methods

### 2.1 Zero-Inflated Gaussian Mixed Models (ZIGMMs)

In a longitudinal microbiome study, we collect *n* subjects and measure each subject at multiple time points *t*_*ij*_, *j* = 1, ···, *n*_*i*_; *i* = 1, ···, *n*. For the *j*-th sample of the *i*-th subject, we denote *c*_*ijh*_ the observed count for the *h*-th taxon at certain taxonomic levels (OTU, e.g. species, genus, classes, etc.). As many previous methods, we analyze one taxon at a time. We first illustrate our model in analyzing the longitudinal microbiome proportion data. We transform the proportions of relative abundance with $\mathrm{arcsine}\left(\sqrt{c_{ijh}/T_{ij}}\right)$, where *T*_*ij*_ denotes the total sequence read. For notational simplification, we denote $y_{ij}=\mathrm{arcsine}\left(\sqrt{c_{ijh}/T_{ij}}\right)$ for any given taxon *h*. For taxa with excessive zeros, it can be assumed that transformed values *y*_*ij*_ may come from either a degenerate distribution having the point mass at zero (zero state) or a Gaussian (i.e., normal) distribution [17]. Thus, the transformed values *y*_*ij*_ can be modeled with the zero-inflated Gaussian distribution:

$$y_{ij}∼\left\{ \begin{array}{l} 0\;\mathrm{with}\;\mathrm{probability}\;p_{ij}\\ N(y_{ij}|\mu_{ij},\sigma^{2})\;y_{ij}\geq 0\;\mathrm{with}\;\mathrm{probability}\;1‐p_{ij} \end{array}\right.$$ (1)

where *μ*_*ij*_ and *σ* are the mean and standard deviation parameters in normal distribution, respectively, and *p*_*ij*_ is the unknown probability that *y*_*ij*_ is from the zero state. The means *μ*_*ij*_ are expressed as:

$$\mu_{ij}=X_{ij}\beta +G_{ij}b_{i}$$ (2)

where *X*_*ij*_ is the vector of covariates for the *j*-th sample of the *i*-th subject; *β* is the vector of fixed effects (i.e. population-level effects), representing the average effects of the covariates over the subjects; *b*_*i*_ is the vector of subject-specific effects, or called random effects, and *G*_*ij*_ is the vector of group-level covariates, which is a subset of the population-level covariates *X*_*ij*_. For longitudinal studies, *X*_*ij*_ could be (1, *X*_*i*_), (1, *X*_*i*_, *t*_*ij*_), or $\left(1,X_{i},t_{ij},X_{i}^{s}t_{ij}\right)$, where $X_{i}^{s}$ is the variable of interest in *X*_*i*_, for example, an indicator variable for the case group and the control group. *G*_*ij*_ could be 1, i.e. only including the subject-specific intercept, or (1, *t*_*ij*_), i.e. including the subject-specific intercept and time effect.

The random effects are assumed to follow a multivariate normal distribution:

$$b_{i}∼N(0,\Psi_{b})$$ (3)

where Ψ_*b*_ is the variance-covariance matrix which can be defined as a general positive-definite matrix accounting for the correlation among the random covariates. In most applications we restrict Ψ_*b*_ to be a diagonal matrix for simplicity.

The zero-inflation probabilities *p*_*ij*_ are assumed to relate some covariates through the logit link function:

$$\mathrm{logit}(p_{ij})=Z_{ij}\alpha$$ (4)

where *Z*_*ij*_ includes some covariates that are potentially associated with the zero state. The simplest zero-inflation model includes only the intercept in *Z*_*ij*_, resulting in the same probability of belonging to the zero state for all zeros. We can also add the random-effect terms into the above model:

$$\mathrm{logit}(p_{ij})=Z_{ij}\alpha +G_{ij}a_{i}$$ (5)

where the random effects *a*_*i*_ are assumed to follow a multivariate normal distribution:

$$a_{i}∼N(0,\Psi_{a})$$ (6)

As an alternative, for longitudinal microbiome count data, we transform the observed count data with *y*_*ij*_ = log_2_(*c*_*ijh*_+1), which equals zero if *c*_*ijh*_ = 0. We assume the *y*_*ij*_ can be modeled with the zero-inflated Gaussian distribution, with the means *μ*_*ij*_ being expressed as:

$$\mu_{ij}=\log(T_{ij})+X_{ij}\beta +G_{ij}b_{i}$$ (7)

### 2.2 The EM algorithm for fitting the ZIGMMs

We propose an EM algorithm to fit the ZIGMMs. We introduce latent indicator variables $\xi =(\xi_{i1},\cdots ,\xi_{in_{j}})$ to distinguish the zero state and the Gaussian state, where *ξ*_*ij*_ = 1 when *y*_*ij*_ is from the zero state and *ξ*_*ij*_ = 0 when *y*_*ij*_ is from the normal distribution. The log-likelihood with the complete data (*y*, *ξ*) is given by:

$$L(\Phi ;y,\xi )=\sum_{i=1}^{n}\sum_{j=1}^{n_{i}}(1-\xi_{ij})\log\left(N(y_{ij}|\mu_{ij},\sigma^{2})\right)+\sum_{i=1}^{n}\sum_{j=1}^{n_{i}}\log\left[p_{ij}^{\xi_{ij}}{\left(1-p_{ij}\right)}^{1-\xi_{ij}}\right]$$ (8)

where Φ represents all the parameters (including random effects) in the ZIGMMs.

The EM algorithm replaces the indicator variables *ξ*_*ij*_ by their conditional expectations ${\hat{\xi }}_{ij}$ (E-step), and then updates the parameters by maximizing $L(\Phi ;y,\hat{\xi })$ (M-step). The conditional expectation of *ξ*_*ij*_ can be calculated as:

$$\begin{array}{l} {\hat{\xi }}_{ij}=p(\xi_{ij}=1|\Phi ,y_{ij})\\ \;=\frac{p(y_{ij}|\mu_{ij},\sigma^{2},\xi_{ij}=1)p(\xi_{ij}=1|p_{ij})}{p(y_{ij}|\mu_{ij},\sigma^{2},\xi_{ij}=0)p(\xi_{ij}=0|p_{ij})+p(y_{ij}|\mu_{ij},\sigma^{2},\xi_{ij}=1)p(\xi_{ij}=1|p_{ij})} \end{array}$$ (9)

If *y*_*i*_≠0, we have *p*(*y*_*ij*_|*μ*_*ij*_,*σ*^2^,*ξ*_*ij*_ = 1) = 0, and thus ${\hat{\xi }}_{ij}=0$.

If *y*_*i*_ = 0, we have

$${\hat{\xi }}_{ij}={\left[\frac{p(\xi_{ij}=0|p_{ij})}{p(\xi_{ij}=1|p_{ij})}p(y_{ij}=0|\mu_{ij},\sigma^{2},\xi_{ij}=0)+1\right]}^{-1}={\left[\frac{1-p_{ij}}{p_{ij}}N(y_{ij}=0|\mu_{ij},\sigma^{2})+1\right]}^{-1}.$$

The parameters in the Gaussian distribution can be updated by fitting a weighted linear mixed model with (1 - ${\hat{\xi }}_{ij}$) as weights:

$$y_{ij}=X_{ij}\beta +G_{ij}b_{i}+{\left(1-{\hat{\xi }}_{ij}\right)}^{-1/2}e_{ij},\;b_{i}∼N_{q}(0,\Psi_{b}),\;e_{ij}∼N(0,\sigma^{2})$$ (10)

If the zero-inflation part does not include the random-effect term, the parameters can be updated by running a binomial logistic regression with ${\hat{\xi }}_{ij}$ as response:

$${\hat{\xi }}_{ij}∼\mathrm{Bin}(1,p_{ij}),\mathrm{logit}(p_{ij})=Z_{ij}\alpha$$ (11)

Otherwise, we can fit the binomial logistic mixed model:

$${\hat{\xi }}_{ij}∼\mathrm{Bin}(1,p_{ij}),\mathrm{logit}(p_{ij})=Z_{ij}\alpha +G_{ij}a_{i},a_{i}∼N(0,\Psi_{a})$$ (12)

The EM algorithm starts from plausible values for the parameters and then updates the parameters as described above until convergence. We use the criterion $\sum_{i=1}^{n}\sum_{j=1}^{n_{i}}[{\left(\eta_{ij}^{\left(t\right)}-\eta_{ij}^{\left(t-1\right)}\right)}^{2}+{\left(\gamma_{ij}^{\left(t\right)}-\gamma_{ij}^{\left(t-1\right)}\right)}^{2}]<\varepsilon \left(\sum_{i=1}^{n}\sum_{j=1}^{n_{i}}\left[{\left(\eta_{ij}^{\left(t\right)}\right)}^{2}+{\left(\gamma_{ij}^{\left(t\right)}\right)}^{2}\right]\right)$ to assess convergence, where $\eta_{ij}^{\left(t\right)}=X_{ij}\beta^{\left(t\right)}+G_{ij}b_{i}^{\left(t\right)}$, $\gamma_{ij}^{\left(t\right)}=Z_{ij}\alpha^{\left(t\right)}+G_{ij}a_{i}^{\left(t\right)}$, and *ε* is a small value (say 10^−5^). At convergence, we obtain the maximum likelihood estimates of the Gaussian-state fixed effects and the associated standard deviations from the final weighted LMM. We then can test H_0_: *β*_*k*_ = 0 according to the LMM framework. We also obtain the estimates of the zero-state fixed effects and the associated standard deviations from the final binomial logistic (or mixed) model. Thus, we can test H_0_: *α*_*k*_ = 0 following the GLM or GLMM framework.

### 2.3 Accounting for within-subject correlations

The weighted linear mixed model (9) restricts the within-subject errors to be independent. We can relax the assumption of independent within-subject errors to account for special within-subject correlation structures:

$$e_{i}=(e_{i1},\cdots ,e_{in_{i}})'∼N(0,\sigma^{2}R_{i})$$ (13)

where *R*_*i*_ is a correlation matrix. Pinheiro and Bates [29] described several ways to specify the correlation matrix *R*_*i*_, for example, autoregressive of order 1, AR(1), or continuous-time AR(1), all of which can be incorporated into our ZIGMMs.

### 2.4 Software implementation

The proposed method has been implemented in the function lme.zig, which is part of the R package **NBZIMM**. In the E-step of the EM algorithm, the conditional expectation of *ξ*_*ij*_ can be calculated as in Eq (9). In the M-step, the parameters in the Gaussian distribution can be updated by repeated calls to the function lme in the R package **nlme** to fit the weighted linear mixed model with (1 - ${\hat{\xi }}_{ij}$) as weights. The other parameters can be updated by repeated calls to the functions glm or glmPQL in the package **MASS** to fit the binomial logistic or mixed logistic model. The function lme is the recommended tool for analyzing linear mixed models. The function lme.zig incorporates the nice features of lme, such as dealing with any types of random effects and within-subject correlation structures. Thus, it provides an efficient and flexible tool for analyzing zero-inflated longitudinal microbiome data. The package **NBZIMM** is freely available from the public GitHub repository http://github.com//nyiuab//NBZIMM.

## 3. Results

### 3.1 Simulation studies

#### 3.1.1 Assess the ZIGMMs in analyzing microbiome proportion data. 3.1.1.1 Simulation design

To evaluate the proposed ZIGMMs, we performed extensive simulations. We first evaluated the ZIGMMs in analyzing microbiome proportion data. We compared ZIGMMs with ZIBR proposed by Chen and Li [25]. We used the function simulate_zero_inflated_beta_random_effect_data in the R package **ZIBR** [25] to simulate longitudinal microbiome proportion data from zero-inflated beta distribution:

$$y_{ij}∼\left\{ \begin{array}{l} 0\;\mathrm{with}\;\mathrm{probability}\;p_{ij}\\ Beta(y_{ij}|u_{ij}\phi ,(1-u_{ij})\phi )\;\mathrm{with}\;\mathrm{probability}\;1‐p_{ij} \end{array}\right.$$

with the link functions logit(*p*_*ij*_) = *Z*_*ij*_*α*+*G*_*ij*_*a*_*i*_ and logit(*u*_*ij*_) = *X*_*ij*_*β*+*G*_*ij*_*b*_*i*_. We employed a case-control longitudinal design with the following settings: 5 time points for each subject, fixed effects in both parts, random intercepts in both parts (i.e. *G*_*ij*_ = 1)). We also considered three numbers of subjects: *n* = 50, 100 and 150, half of which were designated to be cases. We set the regression coefficients as *α* = (*α*_0_, *α*_1_) = (-0.5, 0), *β* = (*β*_0_, *β*_1_) = (-0.5, 0) to test for false positive rate; while *α* = (*α*_0_, *α*_1_) = (-0.5, 0.3),  *β* = (*β*_0_, *β*_1_) = (-0.5, 0.3) to test for power at a low effect setting and *α* = (*α*_0_, *α*_1_) = (-0.5, 0.5),  *β* = (*β*_0_, *β*_1_) = (-0.5, 0.5) to test for power at a high effect setting. The variance of the random effects to control *a*_*i*_ and *b*_*i*_ were set to be 1. The dispersion parameter *ϕ* was set to be 5.

Each simulation was repeated 10000 times. We tested for the hypothesis of *β*_1_ = 0. Empirical power and false positive rate were summarized at the significance level of 0.05. We compared zero-inflated Beta regression mixed model, denoted by ZIBR, and the proposed ZIGMMs with the arcsine square root transformation for proportion data, $\mathrm{arcsine}\left(\sqrt{y_{ij}}\right)$, denoted by ZIGMMs(arcsine), the transformed data was standardized by its standard deviation before model fitting.

**3.1.1.2 Simulation results.** Table 1 shows the comparison of empirical power and false positive rates between ZIGMMs and ZIBR in analyzing the longitudinal microbiome proportion data. ZIGMMs and ZIBR controlled the false positive rates similarly close to the significance level under all three different sample sizes. Although the proportion data were simulated under the zero-inflated beta distribution, ZIGMMs lead to a higher empirical power to detect the group effect than ZIBR.

**Table 1:** False positive rate and power for testing H0: β_1_ = 0 based on ZIGMMs and ZIBR for significance level at 0.05 for various sample sizes.

	False Positive Rate		Power (Low Effect Setting)		Power (High Effect Setting)	
Sample Size	ZIGMMs (arcsine)^†^	ZIBR^‡^	ZIGMMs (arcsine)	ZIBR	ZIGMMs (arcsine)	ZIBR
n = 50	0.0681	0.0577	0.1937	0.1438	0.4100	0.3022
n = 100	0.0554	0.0578	0.3025	0.2218	0.6592	0.5135
n = 150	0.0563	0.0533	0.4308	0.3031	0.8296	0.6906

ZIBR^‡^: Zero-inflated beta mixed model.

ZIGMMs(arcsine)^†^: Zero-inflated Gaussian mixed models with arcsine transformation.

#### 3.1.2 Assess the ZIGMMs in analyzing microbiome count data. 3.1.2.1 Simulation design

We then assessed the ZIGMMs in analyzing microbiome count data. We employed the function sim in **NBZIMM** to simulate zero-inflated longitudinal microbiome count data *c*_*ij*_ as follows. We used the latent-data formulation of the logistic regression to simulate zero-state indicators; the logistic model, *p*(*ξ*_*ij*_ = 1) = logit^−1^(*μ*+*Z*_*ij*_*α*+*G*_*ij*_*a*_*i*_), is approximately equivalent to the model, *u*_*ij*_~*N*(*Z*_*ij*_*α*+*G*_*ij*_*a*_*i*_, 1.6^2^), *u*_*ij*_>*h*⇔*ξ*_*ij*_ = 1 [30], where *h* is a constant determined by the preset overall zero-inflation proportion *p*. Thus, we first simulated latent normal variables *u*_*ij*_ and then set samples with the 100*p*% largest *u*_*ij*_ as from zero state. This method can easily control the overall zero-inflation proportion and also allow for the sample-specific zero-inflation probabilities *p*_*ij*_. For the samples from nonzero state, we simulated counts *c*_*ij*_ from the negative binomial distribution *NB*(*c*_*ij*_|*μ*_*ij*_,*θ*), where *μ*_*ij*_ = log(*T*_*ij*_)+*X*_*ij*_*β*+*G*_*ij*_*b*_*i*_.

We adopted a longitudinal design and utilized four different simulation settings. In all the settings, we generated subjects from two groups (i.e. case or control) and simulated samples at multiple time points for each subject. We considered three numbers of subjects: *n* = 50, 100 and 150, half of which were designated to be cases. Each subject was measured at 5 time points. The random effects, and within-subject correlation structures were set as follows:

1. Setting A: a group variable (β_1_**)** is included as fixed effect in the count part, no fixed effect in the zero-inflation part (i.e. *Z*_*ij*_ = 1), random intercepts in both count and zero-inflation parts (i.e. *G*_*ij*_ = 1)), and no within-subject correlation;
2. Setting B: a group variable is included as fixed effects in both parts, random intercept in the count part only, and no within-subject correlation;
3. Setting C: a group variable is included as fixed effects in both parts, random intercepts in both parts (i.e. *G*_*ij*_ = 1)), and no within-subject correlation;
4. Setting D: a group variable is included as fixed effect in the count part, no fixed effect in the zero-inflation part, random intercept in the count part only, and the within-subject correlation was autoregressive of order 1, AR(1), in the count part;
5. Setting E: a group variable (β_1_**)**, a time variable (β_2_**)**, and a time by main effect interaction term (β_3_**)** are included as fixed effects in both parts, random intercept in the count part only, and no within-subject correlation;

We randomly generated the parameters in the models from reasonable ranges. The parameters to simulate the counts from negative binomial distribution were set by following the work of [31]. This can largely reduce the combinations of parameter values and minimize possible bias from setting inappropriate values for parameters. The ranges were described as follows:

1. To simulate counts similar to real microbiome data, we controlled the means of simulated counts through log(*T*_*ij*_) + *β*_0_, where *β*_0_ is the fixed intercept. We set *β*_0_ = -7 and randomly sampled log(*T*_*ij*_) from the range [7.1, 10.5];
2. For settings A-D, the dispersion parameter *θ* was uniformly sampled from the range [0.1, 5], which yielded highly or moderate over-dispersed counts; for setting E, the dispersion parameter *θ* was set to be 5.
3. To evaluate false positive rates, the fixed effects *β*_1_ was set to be zero. To evaluate empirical powers, we considered two scenarios: a) low effect scenario: *β*_1_ was sampled from [0.2, 0.3]; b) high effect scenario: *β*_1_ was sampled from [0.3, 0.4]; fixed effects in the zero-inflation part were considered in setting B and C, where *α*_1_ was set to be the same as *β*_1_; for setting E, *β*_1_ was set to be equal to *β*_3_. And *β*_2_ was set to be 0 in all scenarios.
4. The random effects *b*_*i*_ and *a*_*i*_ were generated from N(0, τ^2^), for settings A-D, where *τ* was randomly drawn from the range [0.5, 1]; for setting E, *τ* was set to be 0.5.
5. For settings A-D, the overall zero-inflation proportion was set to be chosen from three levels, that is [0, 0.2], [0.2, 0.4] and [0.4, 0.6]; for setting E, the proportion was set to be chosen from [0, 0.5].
6. The correlation coefficient *ρ* and the standard deviation *σ* for AR(1) correlation were both sampled from [0.1, 0.5], and the AR(1) correlation was generated by the function arima.sim from R package **stats**;

The ranges of all the parameters used in the simulation are summarized in Table 2.

**Table 2:** Parameter ranges in simulation studies.

Parameter	Range
log(*T*_*ij*_) + *β*_0_	Unif(0.1, 3.5)
dispersion parameter *θ*	Unif(0.1, 5)
Fixed effects *β*_1_ (false positive rate)	0
Fixed effects *β*_1_ (power)	Unif(0.2, 0.3)
	Unif(0.3, 0.4)
Fixed effect *α*_1_ (Setting B and C only)	Unif(0.2, 0.3)
	Unif(0.3, 0.4)
standard deviation *τ*	Unif(0.5, 1)
correlation *ρ*	Unif(0.1, 0.5)
standard deviation *σ*	Unif(0.1, 0.5)
Overall zero-inflation proportion	Unif(0.0, 0.2)
	Unif(0.2, 0.4)
	Unif(0.4, 0.6)

We repeated the procedure 10000 times for each combination of the parameters. The hypothesis of interest is the fixed effect H_0_: *β*_1_ = 0. Empirical power and false positive rate for testing the hypothesis were calculated at the significance level of 0.05. We compared the proposed ZIGMMs, denoted by ZIGMMs(log), with a previously developed negative binomial mixed model, denoted by NBMMs, and the linear mixed model with the arcsine square root transformed response, $\mathrm{arcsine}\left(\sqrt{y_{ij}/T_{ij}}\right)$, denoted by LMMs.

**Fig 1:**
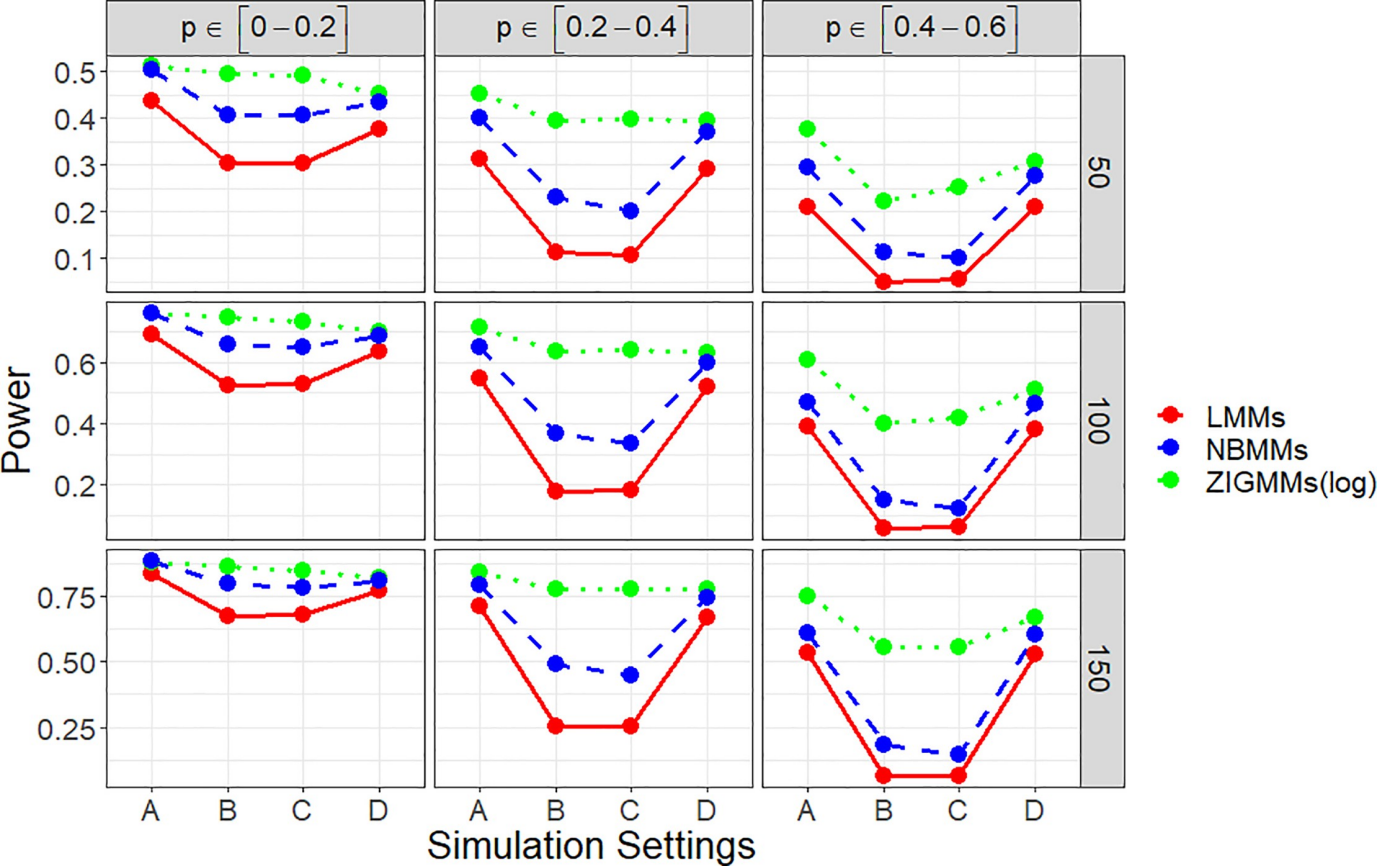
Empirical powers in four simulation settings under low effect scenario.

**Fig 2:**
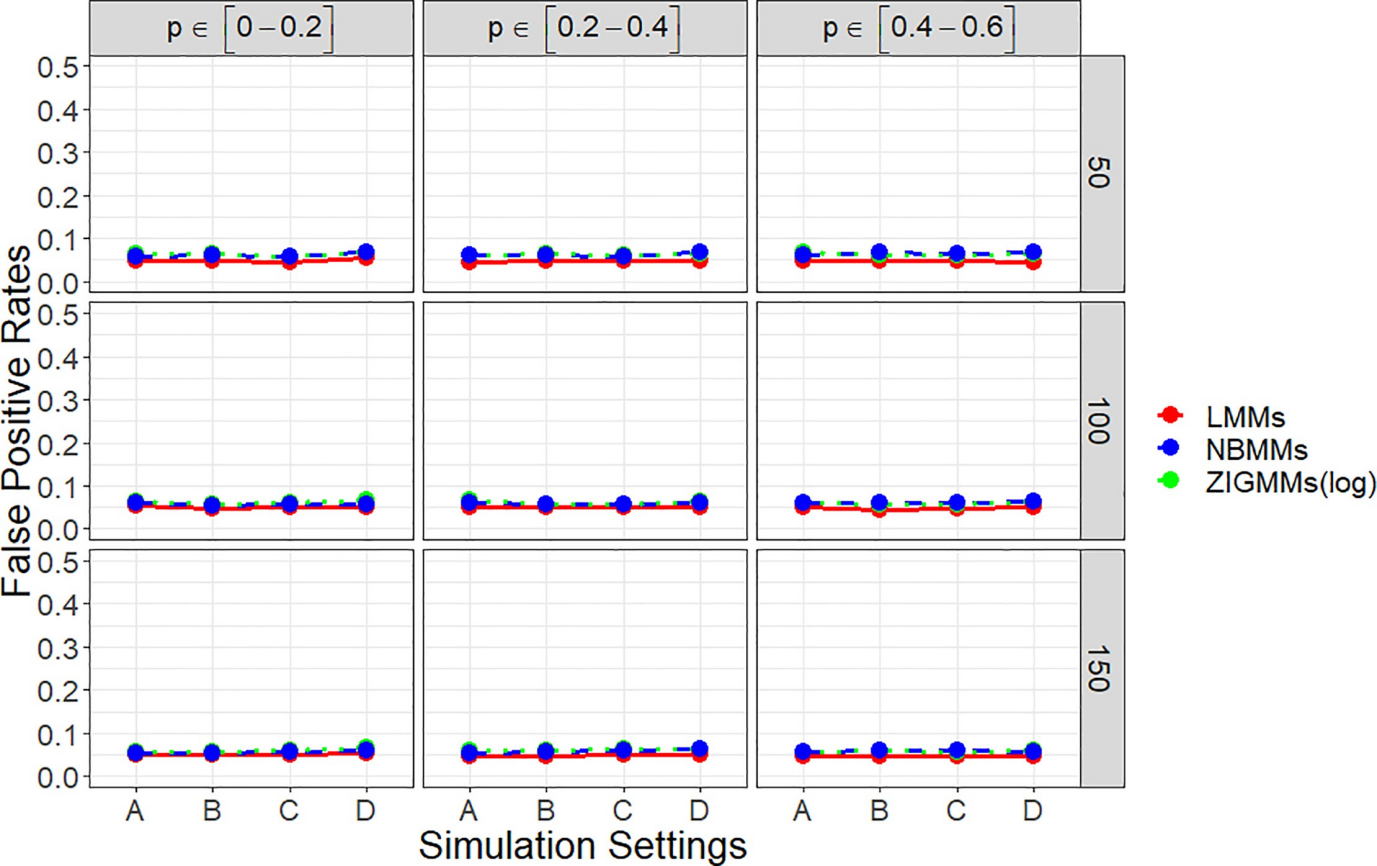
False positive rates in all four simulation settings.

**3.1.2.2 Simulation results.** Fig 1 showed empirical power to detect the group effect for settings A, B, C and D at the low effect scenario. It can be clearly seen that the proposed method performed consistently better than NBMMs and LMMs in all the scenarios. Under setting B and C, we simulated fixed effects in the zero-inflation part. ZIGMMs performed extremely remarkable than NBMMs and LMMs in those two settings, inferring ignoring the association between zero-inflation and any covariate could lead to a significant decrease in power. The power was largely affected by the sample size and the zero-inflation probability. The difference in power among ZIGMMs and NBMMs and LMMs increased significantly as the zero-inflation probability increased. With the zero-inflation proportion less than 20%, ZIGMMs performed similarly as NBMMs but still better than LMMs. ZIGMMs had a more noteworthy higher power than NBMMs and LMMs to detect the fixed effect especially when the data was highly zero-inflated. We also summarized the empirical power to detect the binary group effect for the settings A, B, C and D with the high effect scenario in S1 Fig. In the high effect scenario, ZIGMMs outperformed NBMMs and LMMs more significantly when the zero-inflation probability was higher and the sample size was smaller. Fig 2 displays false positive rates for detecting the group effect. For all the four settings, ZIGMMs controlled the false positive rates close to the significance level under all the combinations of parameters. As expected, the increase in sample size *n* led to the decrease in false positive rates in ZIGMMs.

Table 3 summarized empirical power and false positive rates for setting E comparing LMMs, NBMMs and ZIGMMs. In this setting, we included group variable, time variable and a time by group interaction term in the simulation and reported empirical power and false positive rates for group variable and time by group interaction term. ZIGMMs had a higher power than LMMs and NBMMs for both group effect and interaction term under various sample sizes however ZIGMMs had inflated the false positive rates compared to LMMs and NBMMs especially for the interaction term.

**Table 3:** False positive rate and power for testing H0: β_1_ = 0 and H0: β_3_ = 0 from setting E for significance level at 0.05 for various sample sizes.

	False Positive Rate					
	**Test of β**_**1**_			**Test of β**_**3**_		
Sample Size	LMMs^§^	NBMMs^¶^	ZIGMMs(log)^!^	LMMs^§^	NBMMs^¶^	ZIGMMs(log)^!^
n = 50	0.045	0.053	0.065	0.045	0.064	0.084
n = 100	0.050	0.061	0.067	0.054	0.072	0.082
n = 150	0.047	0.061	0.071	0.050	0.068	0.082
	Power (Low Effect Setting)					
	**Test of β**_**1**_			**Test of β**_**3**_		
Sample Size	LMMs^§^	NBMMs^¶^	ZIGMMs(log)^!^	LMMs^§^	NBMMs^¶^	ZIGMMs(log)^!^
n = 50	0.082	0.158	0.187	0.172	0.251	0.334
n = 100	0.148	0.265	0.325	0.295	0.425	0.563
n = 150	0.204	0.360	0.439	0.405	0.562	0.720
	Power (High Effect Setting)					
	**Test of β**_**1**_			**Test of β**_**3**_		
Sample Size	LMMs^§^	NBMMs^¶^	ZIGMMs(log)^!^	LMMs^§^	NBMMs^¶^	ZIGMMs(log)^!^
n = 50	0.121	0.252	0.304	0.303	0.418	0.558
n = 100	0.224	0.439	0.522	0.507	0.654	0.815
n = 150	0.340	0.602	0.699	0.628	0.769	0.920

LMMs^§^: Linear mixed models.

NBMMs^¶^: Negative Binomial mixed models.

ZIGMMs(log)^!^: Zero-inflated Gaussian mixed models with log transformation.

#### 3.1.3 Assess the computational efficiency of ZIGMMs

To evaluate the computational efficiency of ZIGMMs, we recorded the computation time for ZIGMMs and two other zero-inflated methods in one simulation when sample size is set to be 100. First, we compared ZIGMMs and ZIBR in analyzing the longitudinal microbiome proportion data. We found that the computation time for ZIGMMs and ZIBR in one simulation was 0.011 and 0.023 minutes, respectively. Besides, we compared ZIGMMs and a zero-inflated negative binomial mixed model which was implemented in the R package **glmmTMB** in analyzing the longitudinal microbiome count data, and found that the computation time for ZIGMMs and the zero-inflated negative binomial mixed model in one simulation was 0.009 and 0.041 minutes, respectively. ZIGMMs remarkably outperformed in computational efficiency than the other two zero-inflated methods.

### 3.2 Application to 16S rRNA and shotgun sequencing microbiome data

In our real data analysis, there are two major purposes, one is to evaluate the performances of ZIGMMs in analyzing 16S rRNA data in raw counts, the other is to evaluate the performances of ZIGMMs in analyzing shotgun sequencing data in proportions. So that, we applied our ZIGMMs in two publicly available datasets from Romero, Hassan [24] and Vincent, Miller [32]. Romero, Hassan [24] employed a retrospective case-control longitudinal study to investigate the difference of composition and stability of vaginal microbiota between pregnant and non-pregnant women. They conducted a 16S rRNA gene sequence-based survey among 22 normal pregnant women who delivered at term (38–40 weeks) and 32 non-pregnant women. Vaginal fluid samples were collected every two to four weeks apart for the pregnant group and twice per week for 16 weeks in the non-pregnant group. We analyzed the 16S rRNA sequencing data from Romero, Hassan [24] in terms of counts to evaluate the performances of ZIGMMs(log).

Vincent, Miller [32] used metagenome shotgun sequencing to examine the diversity and composition of the fecal microbiota from 98 hospitalized patients. The prospective cohort study was carried out among 8 patients who were either *Clostridium difficile* infected or colonized and other 90 patients. Clinical data included gender, age, and days from first collection of the fecal samples. The clinical data and shotgun sequencing microbiome relative abundance data were downloaded by R package **curatedMetagenomicData** [33]. The shotgun sequencing data is normally output as proportion data. So, here, we illustrated our ZIGMMs(arcsine) to analyze this shogun sequencing microbiome data from Vincent, Miller [32] in proportions. According to the manual of R package **ZIBR** [26], ZIBR cannot handle missing data. Therefore, we could not compare with ZIBR in our real data example.

We used the following eight different models to compare the performances of LMMs, NBMMs, and ZIGMMs in detecting the dynamic association between host factor and microbiota composition. Models A-D were used in all LMMs, NBMMs and ZIGMMs while models E-G were only used in ZIGMMs:

1. Model A: host factor and time as fixed effects in Gaussian part, random intercept in Gaussian part;
2. Model B: host factor, time, host factor and time interaction term as fixed effects in Gaussian part, random intercept in Gaussian part;
3. Model C: host factor, time, host factor and time interaction term as fixed effects in Gaussian part, random intercept and the within-subject correlation was autoregressive of order 1, AR(1) in Gaussian part;
4. Model D: host factor, time, host factor and time interaction term as fixed effects in Gaussian part, two random effects (i.e., random intercept and time effect) in Gaussian part;
5. Model E: host factor and time as fixed effects only in both zero-inflation part and Gaussian part, random intercept in Gaussian part;
6. Model F: host factor, time, host factor and time interaction term as fixed effects in both zero-inflation part and Gaussian part, random intercept in Gaussian part;
7. Model G: host factor, time, host factor and time interaction term as fixed effects in both zero inflation part and Gaussian part, random intercept and the within-subject correlation was autoregressive of order 1, AR(1) in Gaussian part;
8. Model H: host factor, time, host factor and time interaction term as fixed effects in both zero-inflation part and Gaussian part, two random effects (i.e., random intercept and time effect) in Gaussian part;

The real data and the R code for our analysis are available from the GitHub page: https://abbyyan3.github.io//NBZIMM-tutorial/ZIGMMs-longitudinal.html.

#### 3.2.1 App lication in 16S rRNA longitudinal pregnancy data

We first applied our ZIGMMs to the data of Romero, Hassan [24]. We explored the abilities of ZIGMMs in detecting the dynamic associations between vaginal bacteria taxa composition and two groups (pregnancy vs non-pregnancy) controlled by possible confounding effects of the covariates. We analyzed 16S rRNA sequencing microbiome count data with log transformation (ZIGMMs(log)). In all the eight models, the binary case-control indicator for pregnancy vs non-pregnancy was the host factor of interest (*β*_*1*_), and the collection time (GA_days) was the time variable. An interaction term between host factor and time variable (*β*_*3*_) was included in model B, C, D, F, G and H. We also included age and race as confounding covariates. The sample size was 897 in the final analysis. We included 59 taxa which has a proportion of zeros greater than 0.3 but smaller than 0.9 in our analysis.

Table 4 shows the proportions of significant taxa detected by LMMs, NBMMs and ZIGMMs(log) at the alpha level at 0.05, respectively. The significance of the taxa was evaluated at the alpha level of 0.05 (p-value <0.05) for Models A-H. Test of *β*_*1*_ in Table 4 summarized the proportions of taxa which is significantly differentiated presented between pregnancy group vs non-pregnancy group. Test of *β*_*3*_ in Table 4 summarized the proportions of taxa which is significantly differentiated presented between pregnancy group vs non-pregnancy group over the collection time. The proportions of detected significant taxa in model B, C, D, F, G and H were substantially less than the rates from models A and E. It inferred that the majority of taxa existing in the vaginal microbiome did not possess a time-dependent association between the pregnant and non-pregnant groups. Moreover, it showed that ZIGMMs(log) detected more associated taxa than NBMMs and LMMs. We also found ZIGMMs with fixed effects in zero-inflation and Gaussian part in models E-H decrease slightly in the number of significant taxa detected than ZIGMMs with fixed effects in Gaussian part from models A-D. It implied that those taxa did not possess a strong association between the host factors and the zero-inflation.

**Table 4:** Proportions of significant taxa detected in four models with LMMs, NBMMs and ZIGMMs.

	Model A	Model B		Model C		Model D	
	Test of *β*_*1*_	Test of *β*_*1*_	Test of *β*_*3*_	Test of *β*_*1*_	Test of *β*_*3*_	Test of *β*_*1*_	Test of *β*_*3*_
LMMs^§^	0.29	0.03	0.15	0.03	0.12	0.07	0.10
NBMMs^¶^	0.49	0.12	0.25	0.12	0.25	0.12	0.25
ZIGMMs(log)^!^	0.63	0.34	0.24	0.39	0.27	0.36	0.24
	Model E	Model F		Model G		Model H	
	Test of *β*_*1*_	Test of *β*_*1*_	Test of *β*_*3*_	Test of *β*_*1*_	Test of *β*_*3*_	Test of *β*_*1*_	Test of *β*_*3*_
ZIGMMs(log)	0.54	0.19	0.31	0.20	0.24	0.20	0.20

LMMs^§^: Linear mixed models.

NBMMs^¶^: Negative Binomial mixed models.

ZIGMMs(log)^!^: Zero-inflated Gaussian mixed models with log transformation.

**Fig 3:**
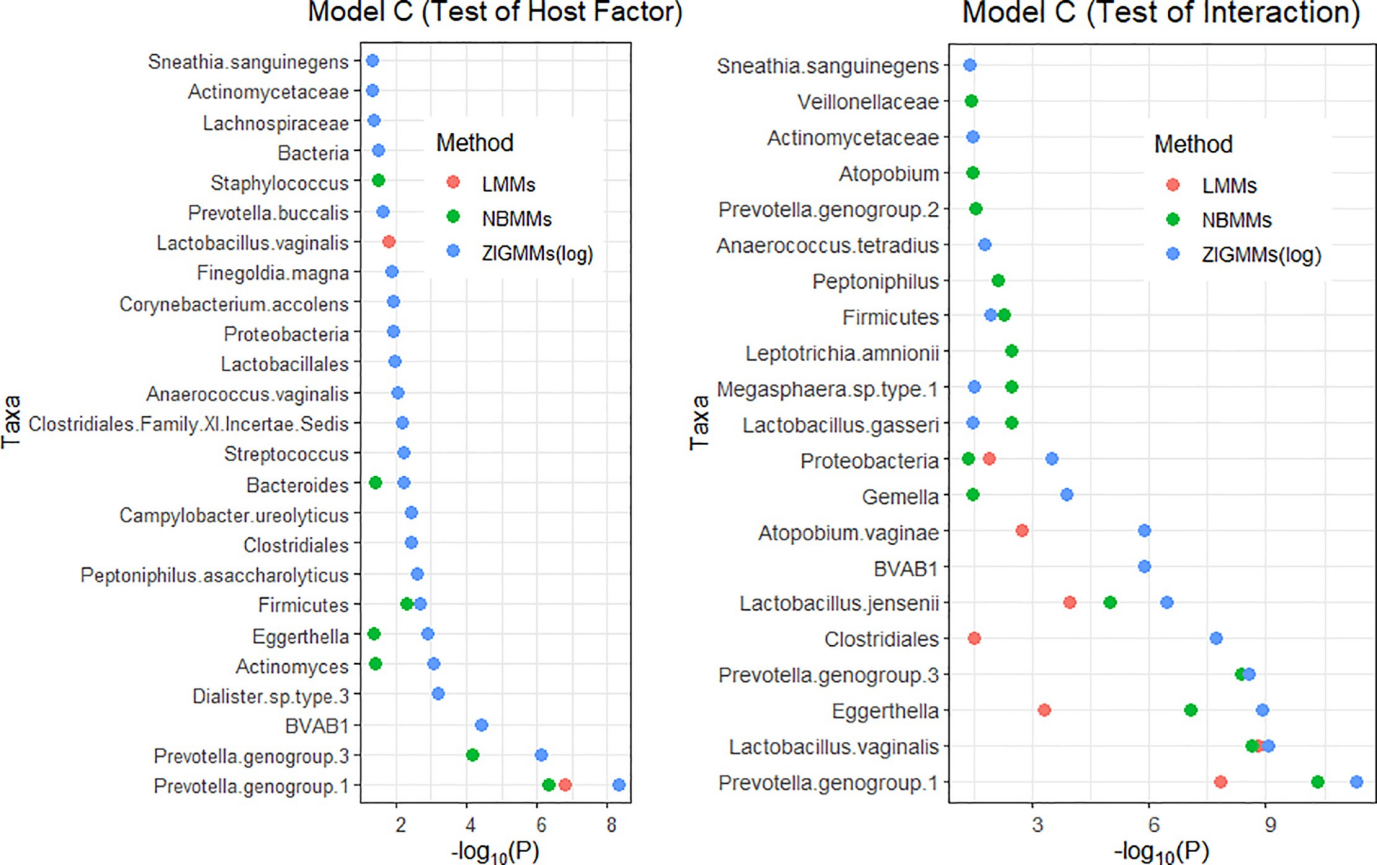
The analyses of ZIGMMs(log), NBMMs and LMMs: minus log transformed p-values for the significant differentially abundant taxa at the 5% significance threshold between pregnancy and non-pregnancy groups for host factor effect (left panel) and interaction effect (right panel) from Model C.

To compare the differences in detecting significant taxa for both host factor and interaction term between LMMs, NBMMs, and ZIGMMs(log), we presented model C in Fig 3 and S2 Fig. Fig 3 shows significant taxa in model C at the 5% significance threshold and minus log transformed p-values for LMMs, NBMMs, and ZIGMMs(log). S2 Fig presents three heatmaps of p-values between the taxa and each variable from model C using LMMs, NBMMs, and ZIGMMs(log). We found that ZIGMMs(log) discovered more taxa than NBMMs and LMMs consistently, and yielded smaller p-values. In model C, we were interested in both the host factor and the interaction effect between time and host factor. ZIGMMs(log) identified not only the same taxa which were detected by LMMs and NBMMs but also more taxa for both effects. For the host factor, several taxa were only identified with ZIGMMs(log), including *Clostridiales*, *Streptococcus*, *Proteobacteria*, *BVAB1* and *Lactobacillales*. For the interaction effect between time and host factor, *Prevotella genogroup 3*, *Gemella*, *Lactobacillus gasseri*, *Megasphaera sp type 1* and *Firmicutes* were identified both by NBMMs and ZIGMMs(log). *BVAB1*, and *Sneathia Sanguinegens* were only identified by ZIGMMs(log). Among them, *bacterial vaginosis associated bacteria 1 (BVAB1)* has been previously reported as a highly specific novel bacteria for bacterial vaginosis in the *Clostridiales* order [34]. Also, the abundance of *Gemella*, *BVAB1*, and *Sneathia sanguinegens* have been reported to change within the duration of pregnancy from another study by Romero, Hassan [35].

#### 3.2.2 Application in shotgun sequencing longitudinal intestinal microbiome data

We then applied our ZIGMMs to the shotgun sequencing microbiome proportion data from Vincent, Miller [32]. In this case, we only compared our ZIGMMs with LMMs. We explored the abilities of ZIGMMs in detecting the dynamic associations between fecal microbiome composition and *Clostridium difficile* colonization or infection. We adapted ZIGMMs in analyzing microbiome proportion data with arcsine transformation (ZIGMMs(arcsine)). In all the eight models, the binary case-control indicator for *Clostridium difficile* colonization or infection vs control was the host factor of interest (*β*_*1*_), and the collection time (days from the first collection) was the time variable. An interaction term between host factor and time variable (*β*_*3*_) was included in models B, C, D, F, G and H. We also included age and gender as confounding covariates. The sample size was 229 in the final analysis. We included 357 taxa which has a proportion of zeros greater than 0.3 but smaller than 0.9 in our analysis.

Table 5 shows the proportions of significant taxa detected by LMMs and ZIGMMs(arcsine) at the alpha level at 0.05, respectively. The significance of the taxa was evaluated at the alpha level of 0.05 (p-value <0.05) for Models A-H. Test of *β*_*1*_ in Table 5 summarized the proportions of taxa which is significantly differentiated presented between *Clostridium difficile* colonization or infection group vs control group. Test of *β*_*3*_ in Table 5 summarized the proportions of taxa which is significantly differentiated presented between *Clostridium difficile* colonization or infection group vs control group over the collection time. We found that our ZIGMMs(arcsine) detected more associated taxa than LMMs in most scenarios. We also found ZIGMMs(arcsine) with fixed effects in zero-inflation and Gaussian part in models E-H increase slightly in the number of significant taxa detected than ZIGMMs(arcsine) with fixed effects in Gaussian part from models A-D. It implied that there is a significant association between the host factors and the zero-inflation in those taxa.

**Table 5:** Proportions of significant taxa detected in four models with LMMs and ZIGMMs.

	Model A	Model B		Model C		Model D	
	Test of *β*_*1*_	Test of *β*_*1*_	Test of *β*_*3*_	Test of *β*_*1*_	Test of *β*_*3*_	Test of *β*_*1*_	Test of *β*_*3*_
LMMs^§^	0.11	0.13	0.12	0.11	0.11	0.10	0.06
ZIGMMs (arcsine)^†^	0.12	0.12	0.19	0.17	0.18	0.11	0.10
	Model E	Model F		Model G		Model H	
	Test of *β*_*1*_	Test of *β*_*1*_	Test of *β*_*3*_	Test of *β*_*1*_	Test of *β*_*3*_	Test of *β*_*1*_	Test of *β*_*3*_
ZIGMMs (arcsine)	0.15	0.14	0.21	0.14	0.23	0.14	0.10

ZIGMMs(arcsine)^†^: Zero-inflated Gaussian mixed models with arcsine transformation.

LMMs^§^: Linear mixed models.

## 4. Discussion

With the emergence of longitudinal microbiome studies, more understandings about the dynamic shifts of the microbiota have been unraveled [8]. It is of interest in studying the dynamic associations between the microbiota and various host factors [8,36]. To realize these research interests, powerful analytic methods are necessary to account for sources of heterogeneity and dependence in microbiome measurements. However, previous methods have not fully addressed the properties of longitudinal microbiome data and are not computationally feasible for analyzing many taxa.

Here, we propose ZIGMMs to model longitudinal microbiome proportion and count data. The method is robust in performance when applied to both 16S rRNA gene sequencing and genome shotgun sequencing data, in terms of proportion or count data. The proportions data, mostly from genome shotgun sequencing data, should be transformed with arcsine square root transformation. For count data, mostly from 16S rRNA platforms, log transformation is more appropriate because if converting those count data to proportion data will lead to very small proportions. The proposed ZIGMMs can effectively handle excessive zeros observed in microbiome data, and can incorporate various types of random effects and within-subject correlation structures [29,37]. We have developed an EM algorithm to fit the proposed ZIGMMs by extending a commonly used procedure for fitting LMMs [37–40]. This allows us to integrate the well-established procedures for analyzing longitudinal data into our ZIGMMs. Our analyses show that our algorithm is efficient and stable for most of the scenarios. We showed the computational efficiency of our EM algorithm by comparing with the other two zero-inflated methods. In the simulations, ZIGMMs outperform LMMs, NBMMs and ZIBR consistently. We have also shown that ZIGMMs can efficiently deal with various fixed and random effects in both normal distribution and zero-inflation models, moreover, and account for the auto-regressive correlation among samples. However, we found ZIGMMs had inflated false positive rates especially in detecting interaction terms, suggesting potential fitting issues. According to Weiss, Xu [41] and Hawinkel, Mattiello [42], most of the parametric methods, such as edgeR, limma–voom and metagenomeSeq, fail to control the false positive rate at the nominal level. A possible reason could be the p-value distributions tend to be smaller than uniform distribution especially when taxa is highly inflated [42]. Thus, in current analysis of a real microbial data, researchers normally focus on the top abundant taxa with less zero-inflation rates.

Moreover, we applied our method to two previously published datasets and compared the performances of LMMs, NBMMs and ZIGMMs in detecting the dynamic association between host factor and taxa composition. We could not apply the ZIBR in the real data since according to the manual of R package ZIBR, it could only deal with subjects measured at the same number of time points [26]. We found that our ZIGMMs was capable to detect more significant taxa than LMMs and NBMMs. The differences between our ZIGMMs and the other two methods were more substantial when analyzing the taxa with high zero rates. Notably, we found that several taxa from Romero, Hassan [24], which have only been identified by ZIGMMs, have been previously reported for the associations between pregnancy and vaginal bacterial composition by Romero, Hassan [35]. However, we still encounter the fitting issues similarly as other parametric methods to control false positive rates under nominal level, especially when analyzing complex microbiome/metagenomics data. A future plan is to develop analyzing methods under Bayesian framework using MCMC algorithm to possibly address the current fitting issues.

## Supporting information

S1 Fig Empirical power of hypothesis in four simulation settings under high effect scenario. (PDF)

S2 Fig Heat map for p-values between the taxa and each variable from Model C using LMMs (left panel), NBMMs (middle panel) and ZIGMMs (right panel). The sign “+” indicates the positive effect.
(PDF)
