# Supplementary figures and images for: Zero-Inflated gaussian mixed models for analyzing longitudinal microbiome data
Source: PLoS One. 2020 Nov 9;15(11):e0242073. doi: 10.1371/journal.pone.0242073 (PMC7652264; doi:10.1371/journal.pone.0242073)

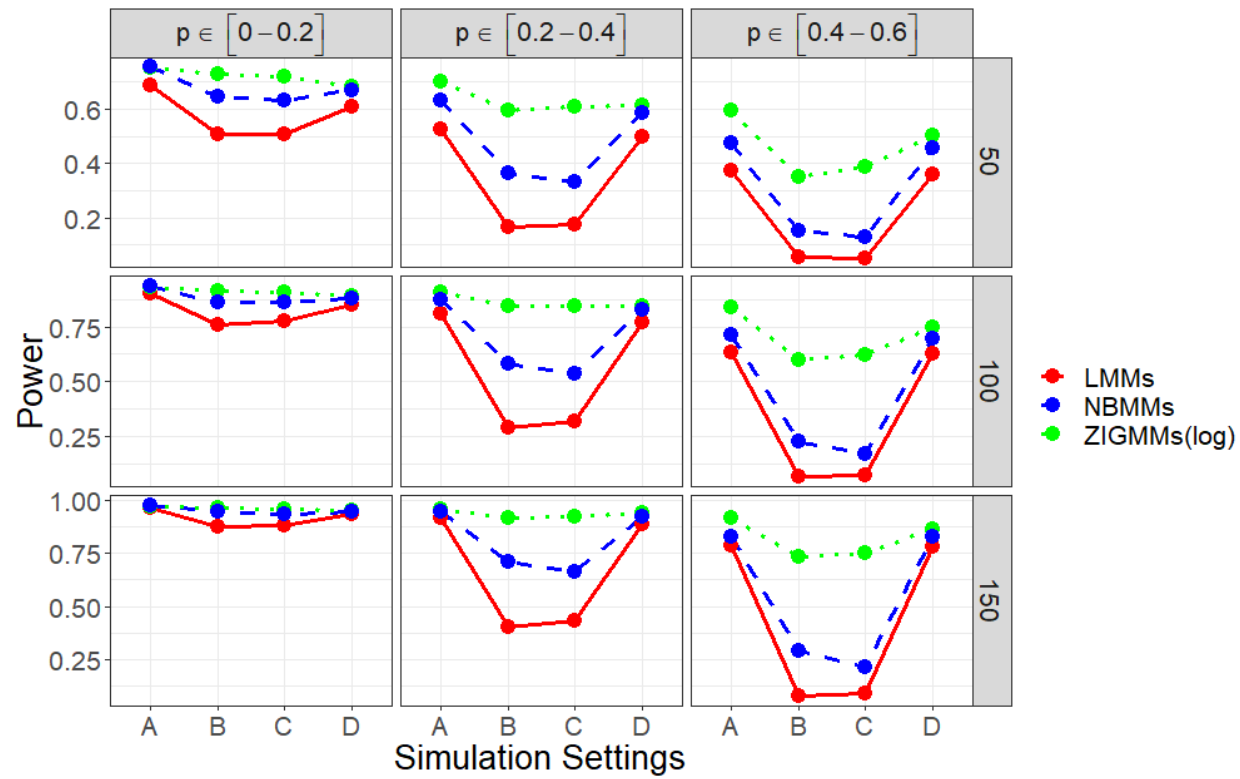

**Fig A.1** Empirical power of hypothesis in four simulation settings under high effect scenario

Supplement: S1 Fig — (PDF) [file pone.0242073.s001.pdf]
