# Supplementary material for: Zero-Inflated gaussian mixed models for analyzing longitudinal microbiome data
Source: PLoS One. 2020 Nov 9;15(11):e0242073. doi: 10.1371/journal.pone.0242073 (PMC7652264; doi:10.1371/journal.pone.0242073)
Supplement: S2 Fig — Heat map for p-values between the taxa and each variable from Model C using LMMs (left panel), NBMMs (middle panel) and ZIGMMs (right panel). The sign “+” indicates the positive effect. (PDF) [file pone.0242073.s002.pdf]

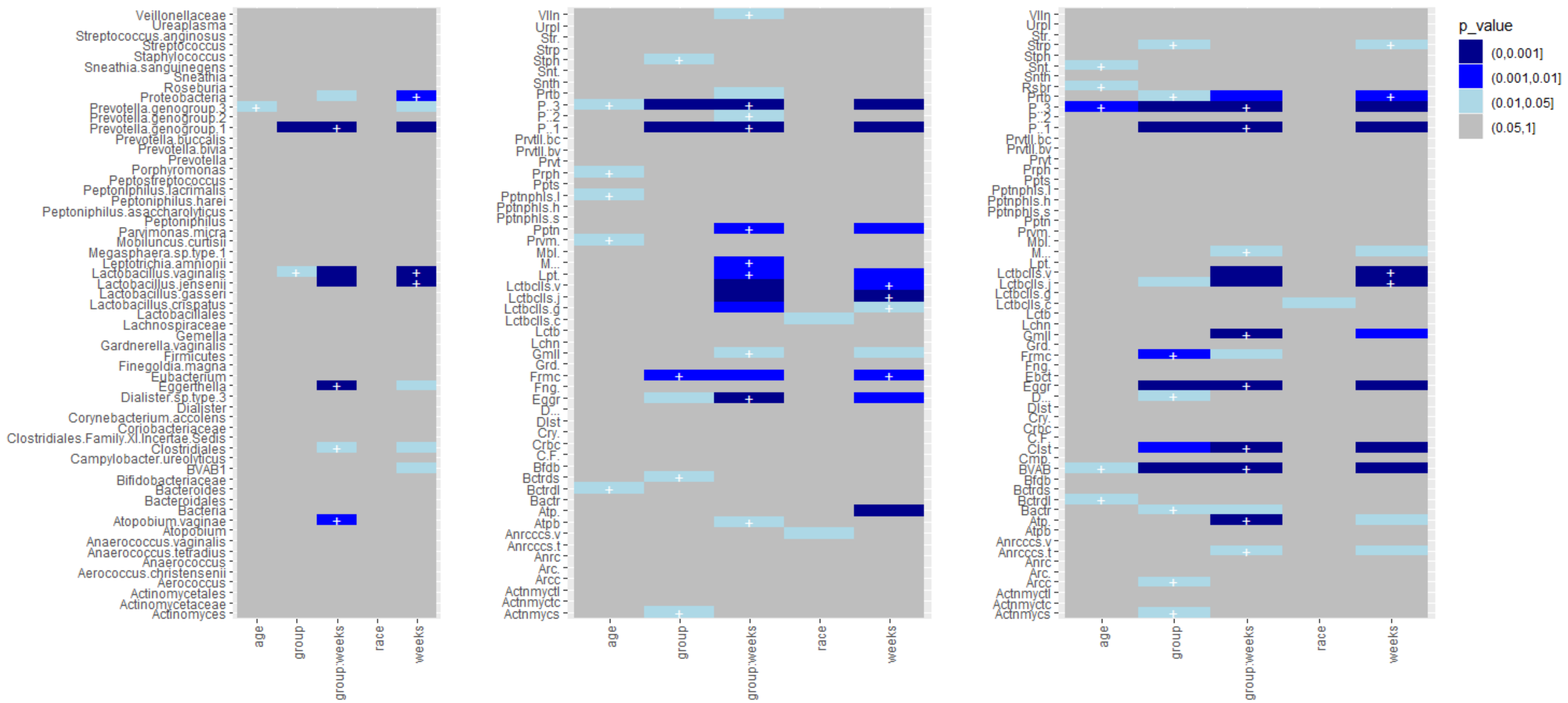

Fig A.2 Heat map for p-values between the taxa and each variable from Model C using LMMs (left panel), NBMMs (middle panel) and ZIGMMs (right panel). The sign "+" indicates the positive effect.
